# 

**Published:** 1842-11-05

**Authors:** 


### CONTENTS OF No. 45.

Dr. West's Case of Malformation of the Heart and Great Vessels, attended with Cyanosis; with remarks upon this affection, - 705

ANALECTA: The Surgical Practice at the Hôtel Dieu, - - - - - 709

M. Bourgery on the minute Structure of the Lungs in Man and the Mammalia, - - - - - - - - - 711

Dr. Negrier on a very simple means of arresting Epistaxis, - 712

Medicinal Properties of Bromine and its Compounds, - 712

M. Piorry on a peculiar granular appearance of the Buffy Coat of the Blood, - - - - - - - - - - 717

M. Berard's Case of Intra-Parietal Hernia after a wound of the Abdomen, - - - - - - - - - - 717

M. Dumeril on the Preservation of the Nitrate of Silver, - 718

M. Blandin's Case of Exophthalmia, &c., - - - - - 718

Preparation of Mercurial Ointment, - - - - - - 719

Dr. Teale's Case of Strangulated Ventral Hernia, - - 719

Subconjunctival Operation for Cataract, - - - - - 720

Quinine found in the Urine and in the Blood, - - - 720

Cryptogamia in the Roots of Hairs, - - - - - - 720

